# Efficacy of personalized feedback in encouraging sustainable washing behavior: evidence from a pilot study in Germany

**DOI:** 10.3389/fpsyg.2024.1473953

**Published:** 2025-01-28

**Authors:** Laura Höpfl, Ivan Đula, Francisco Kiss, Rebecca Walter, Maria Wirzberger

**Affiliations:** ^1^University of Stuttgart, Teaching and Learning with Intelligent Systems, Stuttgart, Germany; ^2^Robert Bosch GmbH, Stuttgart, Germany; ^3^University of Stuttgart, Cluster of Excellence EXC 2075 'Data-Integrated Simulation Science', Stuttgart, Germany; ^4^University of Stuttgart, Interchange Forum for Reflecting on Intelligent Systems, Stuttgart, Germany

**Keywords:** behavioral science interventions, barriers in the real world, individualized feedback, personalized interventions, energy consumption reduction

## Abstract

**Introduction:**

Reducing household energy consumption through behavioral changes is a key strategy in addressing the emissions driving the climate crisis. Behavioral changes in affluent households toward more sustainable practices can have a significant positive impact. Prior research highlighted the role of individual values and motivational factors in shaping sustainable clusters. A more personalized approach toward encouraging the resulting clusters of people to adopt more sustainable strategies seems promising. Such an approach could incorporate aligned feedback, which has been proven to be a powerful mechanism throughout learning processes.

**Method:**

Over 9 weeks, a pilot study with 50 participants investigated the impact of different types of feedback on washing behavior. The within-subjects design included (1) a baseline condition, (2) feedback on energy consumption (kWh), and (3) feedback on monetary costs per cycle (EUR). Data collection encompassed pre- and post-condition surveys, a final comprehensive survey, and a diary-formatted table. The primary objective was to evaluate the potential for individualization. Asynchronous structured interviews were conducted at the end to explore participants' perceptions and washing behaviors.

**Results:**

While we found effects for the feedback manipulation, we found no differences between user clusters in individual washing behaviors. Furthermore, participants qualitatively reported habitual changes, feeling more knowledgeable about the monetary impacts of specific washing programs and temperatures, and wished for a more accessible preset time function. Most participants expressed willingness to switch to a dynamic energy price if it translated to significant cost savings.

**Discussion:**

Our findings may support the notion that individualized behavior change strategies are promising. In general, these strategies should be easily applicable, cost-effective, and promote habits to be exerted regularly. Arising methodological limitations suggest further research in this domain. From an applied perspective, our research provides valuable insights for designing products, services, and regulations by governments and companies, empowering them to develop more effective strategies for reducing energy consumption.

## 1 Introduction

Reducing greenhouse gas (GHG) emissions is essential to decelarate climate change and mitigate its unpredictable negative consequences (Lee, 2008; Grubb et al., 2022). GHG emissions are measurable and can be influenced by human activities. Examples of reduction strategies include utilizing low-carbon energy sources and carriers, adopting energy-efficient appliances and systems, and promoting behavioral changes to decrease energy demand (Creutzig et al., 2022). Since private households are a significant source of GHG emissions, behavioral changes can substantially reduce those emissions (Dhakal et al., 2022). In general, GHG emissions of high-income households are typically above average due to larger homes and more frequent air travel enabled by greater resource access. On a global scale, households within the highest 10% income bracket are responsible for ~36–45% of the total GHG emissions (Shukla et al., 2022).

Thereby, heating is an essential component that significantly contributes to household emissions across many countries. While in regions such as Brazil or India, cold washes are the norm (Spencer et al., 2015), utilizing less energy, in most of Western Europe, washing clothes and textiles with heated water ist common (Pakula and Stamminger, 2010). Consequently, the share of electricity consumption for laundry in Europe represents between 3.8 and 9.2%, thereby contributing to household emissions on a daily basis. Influencing consumer preferences and demand is essential for driving change among high-income households. Therefore, we investigate whether technical design features in consumer products can support behavioral adaptation toward more sustainable energy consumption.

Consequently, addressing consumer preferences and demand is critical to achieving a change for high-income households. Entrepreneurs and companies can shape consumer preferences by designing their products, systems, and services with reduced energy demand and fostering a more sustainable usage. For companies, a shift toward more sustainable design is both an opportunity and a potential source of conflict. Opportunities lay, for example, in lowering the dependence on environmentally critical resources (Bansal, 2005; Lubin and Esty, 2010), which could be pivotal for product manufacturing. Additionally, a more sustainable product design can reduce resource consumption in both production and subsequent consumption phases, for example, through up-scaling interventions targeting behavior change. Furthermore, companies can leverage the growing demand for sustainable products and services by offering environmentally friendly and socially responsible products. This trend presents an opportunity for companies to develop competitive advantages that align with the values and preferences of environmentally conscious consumers, potentially increasing the market share in this segment.

Existing evidence indicates various approaches that can promote sustainable behavior through design. Concretely, product designers could leverage the effects of social influence (Albarracín et al., 2024), habit formation (Albarracín et al., 2024), feedback (Hummel and Maedche, 2019), emotional attachment toward a product, tangibility, ease of use (White et al., 2019), boosting (Hertwig, 2017), or nudging (Hummel and Maedche, 2019). Boosting involves empowering people to engage in well-informed independent decision-making (Hertwig, 2017), whereas nudging seeks to influence people's conscious and unconscious decisions by altering the choice architecture (Balmford et al., 2021). Thereby, a nudge is defined as an external factor that can influence people's behavior (Thaler and Sunstein, 2008). These subtle impulses aim to guide individuals toward desired behaviors. For instance, positioning healthy food options at eye level in a cafeteria can encourage consumers to make healthier choices (Vandenbroele et al., 2020).

Comprehensive comparative research has shown significant effects related to behavior change for several interventions. These interventions include providing feedback to the user, setting a default for the task to encourage preferred behavior, simplifying the task to reduce effort, and adding a comparison through social reference (Hummel and Maedche, 2019). Looking into established mechanisms of behavioral change in learning research, we see that particularly feedback is a powerful tool that has been employed across various formal and informal learning and training settings (Hattie and Timperley, 2007; Shute, 2008). Feedback can convey valuable insights for users, including achievements, consequences, and associated implications, such as financial and environmental implications.

To ensure that feedback is actionable, Hysong et al. (2006) consider timeliness, personification, customizability, and non-punitiveness as crucial factors. Thereby, non-punitiveness involves avoiding a punishing feedback tone. Individualization refers to feedback on personal performance rather than aggregated data. Timeliness concerns the frequency of feedback, while customizability addresses presenting performance data in a personally meaningful way (Hysong et al., 2006). Considering its demonstrated effectiveness as tool to facilitate learning processes in numerous studies, feedback holds promise for facilitating the adoption and enhancement of sustainable behaviors among individuals. Even though behavioral interventions, such as feedback, are effective, the challenge remains that they often exhibit modesty due to temporary, context-dependence (Balmford et al., 2021). Hence, behavior change could be influenced by addressing individual differences with personification such as monetary incentivization through feedback. Individualized products and services might have great potential since they can cater to users' diverse needs, motivations, and values (Koren et al., 2015).

Previous research indicated that incorporating personal attitudes, values, and motivational factors could be an effective strategy for promoting sustainable thinking and behavior (Steg and de Groot, 2019). More precisely, developing personalized approaches for encouragement might be promising (Höpfl et al., 2024; Briem et al., 2019). Working toward this goal, previous research builds on dimensions such as belief in climate change, collaboration, or skepticism concerning sustainability (Höpfl et al., 2024). Based on a sample of 351 participants, the proposed approach identified five intention-based sustainability clusters via data-driven clustering. Individuals assigned to the Socially sustainable cluster are characterized by peer group-motivated pro-environmental behavior, while those assigned to the Responsible savers cluster emphasize sustainable products motivated by environmental concerns. In contrast, individuals assigned to the Unconcerned spenders cluster display a greater need for immediate gratification, and those assigned to the Comfort-oriented cluster have a low awareness of sustainable consumption. Lastly, individuals assigned to the Skeptical consumer cluster exhibit the highest barrier to exerting a more sustainable lifestyle. Embedding such distinctive perspectives and related behavioral implications in sustainability research enables a more comprehensive understanding of individuals' willingness and motivation to change.

Consequently, building on the introduced sustainability clusters, we further explore the potential of personalized behavioral interventions to promote reduced GHG emissions and encourage a less carbon-intense lifestyle. Utilitzing a mixed-methods approach that combines behavioral measurements and interviews within a field study, we investigate strategies to promote behavioral changes in private households, focusing on washing practices as a case example. Sustainable washing behavior can be characterized by variables such as washing at lower temperatures (Hauthal, 2012), longer duration (Alborzi et al., 2017), and lower counts of wash loads (Pakula and Stamminger, 2010). Furthermore, longer washing cycles correlate with the lower temperatures (Alborzi et al., 2017).

We consider feedback a fundamental strategy for promoting behavioral change due to its evidence-based effects, informative value in design, and flexibility in adjusting various variables. Consequently, we analyze different feedback conditions and their impact on behavior in a field study conducted in German households. More precisely, these include (i) information on kilowatt-hours spending, (ii) monetary investment, and (iii) a baseline without further information. Based on evidence from previous research (Höpfl et al., 2024), we investigate three core hypotheses^1^: First, we propose that participants will exert different washing behaviors if they receive the outlined different types of feedback (H1). Additionally, participants assigned to different sustainability clusters should exert different washing behaviors (H2). Lastly, we assume that participants assigned to different sustainability clusters will respond with different washing behaviors to feedback conveyed in euro [EUR, €] or kilowatt-hours [kWh] (H3). We assume the monetary factor to be the stronger determinant (Vohs et al., 2006) compared to energy consumption.

Taken together, while we found effects for the feedback manipulation, we found no differences between user clusters in individual washing behaviors. Furthermore, participants qualitatively reported habitual changes, feeling more knowledgeable about the monetary impacts of specific washing programs and temperatures, and wished for an adaption of specific washing modes, such as a more accessible preset time function. Most participants expressed willingness to switch to a dynamic energy price if it translated to significant cost savings. Our findings support the notion that individualized behavior change strategies might be promising. In general, these strategies should be easy, cost-effective, and promote habits to be exerted regularly. Arising methodological limitations suggest further research in this domain. From an applied perspective, our research provides valuable insights for designing products, services, and regulations by governments and companies, empowering them to develop more effective strategies for reducing household energy consumption.

## 2 Methods

In a pilot study, we investigated whether there are variations in behavioral reactions to modified feedback among different sustainability clusters. In addition to a 9-week quantitative behavioral field experiment phase, we conducted structured asynchronous interviews (*n* = 39) to receive further information about the perception of the different feedback types. The study plan for data collection and analysis was approved by the Committee for Responsibility in Research of Stuttgart (approval number: Az. 23-061) and pre-registered at OSF.^2^ We obtained informed consent from all participants and followed the General Data Protection Regulation (GDPR; EU, 2016). Furthermore, we closely followed relevant guidelines and regulations outlined in Standard 8 of the Ethical Principles and Code of Conduct for Psychologists (American Psychological Association, 2010).

### 2.1 Participants

Most households in Germany fall within the top 10% of the highest income bracket worldwide, making the sample suitable for our purposes. To accurately represent the 80 million German inhabitants responsible of washing, we utilized the services of TestingTime (2023), a specialized agency known for selecting representative samples. Thereby, an initial set of 51 participants was selected (see text footnote 2). As part of the screening process, participants were asked to identify the household items they personally use on a regular basis. Those who did not report regular use of a washing machine were excluded from the study. After data cleaning and excluding a participant due to missing data, our final sample size consisted of 50 participants aged between 24 and 66 (*M*_*age*_ = 42.4 years, *SD*_*age*_ = 8.73) with a majority of 66% being female. Most participants resided in either 3-person households (43.14%) or 4-person households (43.14%) and reported having one child (52.94%) or two children (41.18%) residing in the same household. Regarding monthly household income, most participants fell between 3,600 and 5,000 € (43.14%) or exceeded 5,000 € (39.23%). Participants received a monetary compensation of 95 € for completion of the study.

### 2.2 Design

We implemented a within-subjects design, wherein each participant underwent three feedback conditions for 3 weeks each, resulting in different information displayed on an electricity meter. The study explored the impact of sustainability clusters (Socially sustainable cluster vs. Responsible savers cluster vs. Unconcerned spenders cluster vs. Comfort-oriented cluster vs. Skeptical consumer cluster) and feedback (baseline vs. energy consumption in kWh vs. monetary investment in EUR) as independent variables. The dependent variables in this study encompass various aspects of washing behavior, such as temperature, count of wash loads, and cycle duration in minutes. The three conditions were presented sequentially in a fixed order, as we assumed that the monetary investment would have a greater impact and that the effects of energy consumption would disappear. The qualitative portion of the study involved conducting structured interviews. A specific interview guide was used to maintain consistency across interviews, with open-ended questions focusing on feedback conditions, changes in washing behavior, and the delay function of washing machines. We selected an asynchronous format to ensure consistent question delivery, give participants the flexibility to respond at their convenience, and allow for more thoughtful and detailed answers.

### 2.3 Materials

**Figure 1:**
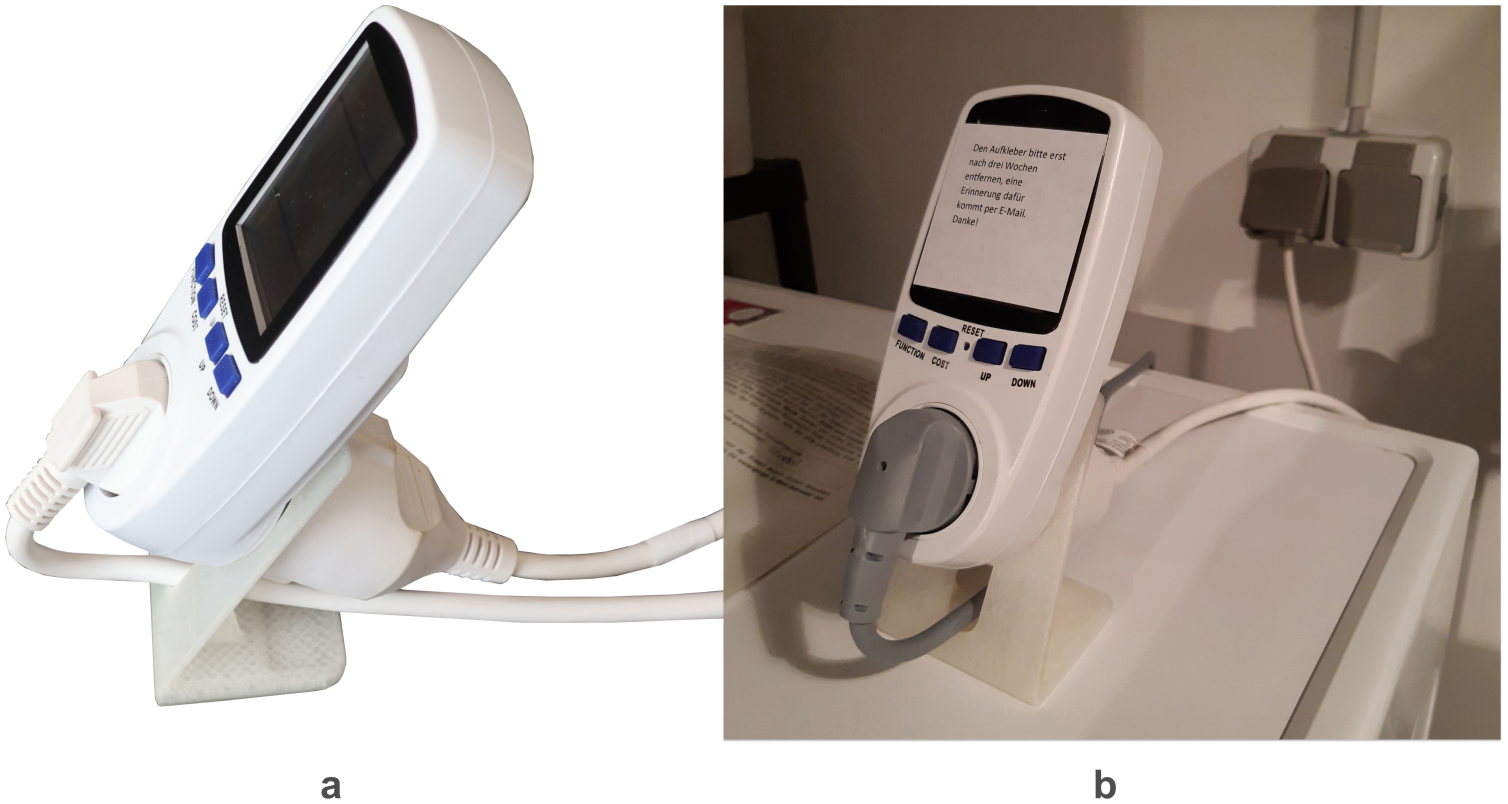
Experimental setup used across the 9-week behavioral field experiment. **(A)** Displays the standard setup, consisting of an energy meter connected to the cable of the washing machine, an extension cable, and a stand. **(B)** On the right shows an exemplary setup in a participant's home with the sticker on the front in place during the baseline condition.

**Figure 2:**
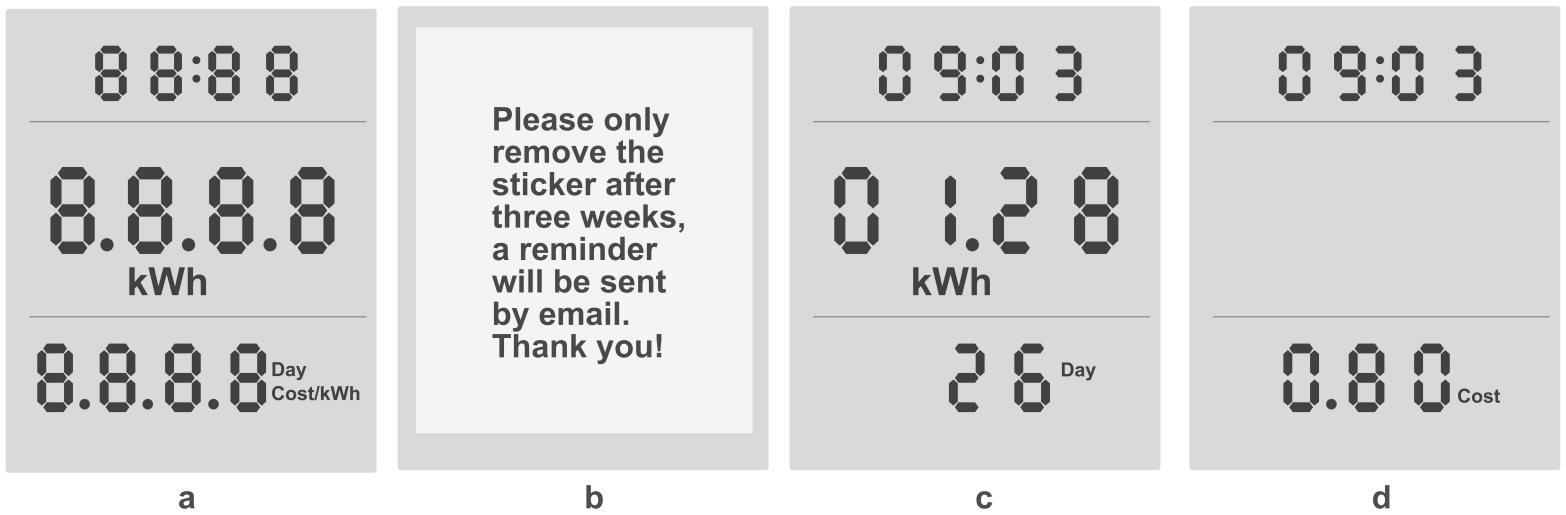
Visual setup of conditions. Setup of **(A)** all displayable options of the energy meter, **(B)** baseline condition (sticker in place), **(C)** condition 2 (time, kWh, day), **(D)** condition 3 (time, cost per wash cycle).

Visual feedback was provided on electricity meters of the brand X4-LIFE, which were chosen due to their versatility. All materials were sent via mail to each participant's home, including the operating instructions, an extension cable, a stand for the appliance, instructions for assembly, data protection information, participant information, and a table for documenting the washing cycles (see text footnote 2). The experimental arrangement is depicted in Figure 1, alongside the setup used by an individual participant. In Figure 2, the three different conditions are dispalyed. The study included four surveys and utilized the online survey tool SoSci Survey (Leiner, 2019) for data collection. Additionally, a self-created diary-formatted table was utilized to record washing behavior.

Assessing participants' affiliation with sustainability clusters involved a previously developed screening instrument derived from Höpfl et al. (2024). The instrument builds on established trust scales regarding sustainable labels (Voon et al., 2011), skepticism toward pro-environmental advertising (Mohr et al., 1998; Obermiller and Spangenberg, 1998), economic benefits in terms of pricing, social status, and social norms (Lee, 2008; Suki and Suki, 2015), believe in human-made climate change, and care for sustainability (see text footnote 2).

### 2.4 Procedure

**Figure 3:**
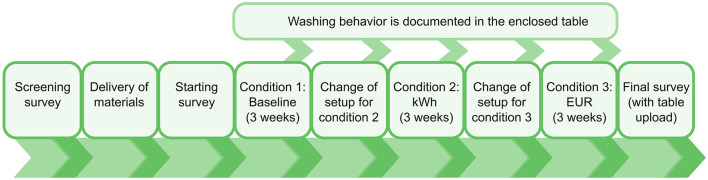
Procedure of the experiment. Depicted is the flow of tasks a user has to perform to complete the study.

The data collection process included an initial survey, a survey administered after each condition, a concluding survey, a diary-formatted table for ongoing entries, and a reflective interview. The experimental procedure is depicted in Figure 3. The instructions and survey links were sent to the participants via email. Data collection was conducted utilizing the SoSci Survey platform (Leiner, 2019). Participants completed the first survey after receiving the required hardware via mail and installing the device at their washing machines. Additionally, they provided demographic data, the cost of 1 kWh in their respective households, which laundry cycles they used, and filled in the screening for assignment to a specific sustainability cluster. Over 9 weeks, participants meticulously documented their washing behavior in a printed table designed to be affixed to the washing machine. Setup changes related to different conditions were performed upon instruction by removing stickers on the energy meter or changing the setup of the energy meter. In condition (1), the baseline condition, the energy meter was sealed with a sticker, and participants received no further information. Participants annotated the date, time, name of the laundry cycle, temperature, cycle duration in minutes, time preselection (in h), and individual time flexibility for certain wash cycles in the provided table in weeks 1–3. In kWh condition (2), participants removed the sticker and got feedback in the form of kWh per wash cycle. In addition, participants annotated the feedback in the form of kWh in weeks 4–6. In EUR condition (3), participants received feedback in form of EUR, which was calculated as the product of the consumed kWh and the individual energy price per kWh. In addition, participants annotated the EUR per washcycle in weeks 6–9.

After completing the three conditions, the participants received the structured interview questions via email to answer asynchronously. They submitted their responses by sending audio files via email. In the final questionnaire, participants confirmed conscientious and responsible participation and provided information on unusual events during participation, such as extended visits, illness, vacation, or other. Participants needed, on average, *M*_*duration*_ = 35 min (*SD*_*duration*_ = 10.25, range: 17–55 min) to fill in all four surveys and conduct the required setup changes.

### 2.5 Data preparation and scoring

Certain participants were unable to provide the correct participant code consistently, necessitating the establishment of a mapping for these cases. Sustainable washing behavior was measured by cycle count, temperature, and cycle duration in minutes, which were calculated from the washing tables participants provided. We employed a decision tree to assign participants to different sustainability clusters, following the approach reported in Höpfl et al. (2024). We assessed the results using the silhouette score for cluster evaluation. The procedure resulted in clusters of differing sizes; the Skeptical consumer cluster included nine participants, no participant was clustered according to the Socially sustainable cluster, the Responsible savers cluster included 21 participants, the Unconcerned spenders cluster included two participants, and the Comfort-oriented cluster included 16 participants. The data preparation, scoring, and analysis code was written in Python 3 (van Rossum and Drake, 2009), with Anaconda 3 (Anaconda Inc., 2020), and Jupyter Notebook (Kluyver et al., 2016) as integrated development environment (IDE) as well as Scipy (Virtanen et al., 2020) and Scikit-learn (Pedregosa et al., 2011) as involved libraries. The interviews were transcribed verbatim and analyzed using MAXQDA (MAXQDA, 2021), a software designed to assist in coding.

## 3 Results

While we found differences in the feedback manipulation, we found no effect between user clusters in individual washing behaviors. Furthermore, participants qualitatively reported habitual changes, feeling more knowledgeable about the monetary impacts of specific washing programs and temperatures, and wished for a more accessible preset time function. Most participants expressed willingness to switch to a dynamic energy price if it translated to significant cost savings.

### 3.1 Quantitative results

On average, participants washed *M*_*cyclecount*_ = 44.39 times throughout the experiment (*SD*_*cyclecount*_ = 26.85, range: 12–157 times), breaking down to 4.93 cycles per week. The average duration was *M*_*duration*_ = 106.13 min per wash load (*SD*_*duration*_ = 34.78, range: 14–257 min). The mean temperature over all participants was *M*_*temperature*_ = 41.36°C (*SD*_*temperature*_ = 4.60°C, range: 30–56.35°C). The clustering did not perform well on the current study's data, with a silhouette score of *s* = 0.24. Descriptive characteristics of washing behavior across sustainability clusters are displayed in Table 1, whereas Table 2 outlines sustainability-related values, motivations, and attitudes means across these clusters.

**Table 1:** Descriptive statistics for washing behavior across sustainability clusters.

**Cluster**	**Temperature**		**Cycle length (min)**		**Cycle count**	
	*M*	*SD*	*M*	*SD*	*M*	*SD*
Skeptical consumer	43.79	9.84	96.47	50.78	59.86	24.45
Responsible savers	40.66	10.63	101.37	60.67	75.18	44.30
Unconcerned spenders	36.71	4.73	117.5	62.68	39.11	11.35
Comfort-oriented	40.39	14.52	113.03	55.52	51.47	19.95

Skeptical consumer cluster (10 participants), Responsible saver cluster (22 participants), Unconcerned spenders cluster (two participants), and Comfort-oriented cluster (17 participants).

**Table 2:** Dimensions of sustainability-related values and attitudes across clusters.

**Sustainability clusters**	**Social status**		**Skepticism**		**Economic benefit**		**Trust**		**Care sustainability**	
	*M*	*SD*	*M*	*SD*	*M*	*SD*	*M*	*SD*	*M*	*SD*
Skeptical consumer	2.65	0.56	2.20	0.39	2.50	0.67	2.93	0.52	1.40	0.52
Responsible savers	3.18	0.81	3.26	0.70	2.98	0.65	4.29	0.36	1.00	0.00
Unconcerned spenders	1.67	0.94	1.83	1.18	2.00	1.42	2.50	2.12	3.50	0.71
Comfort-oriented	2.59	0.59	3.02	0.57	2.44	0.68	3.90	0.28	2.00	0.00

Skeptical consumer cluster (10 participants), Responsible saver cluster (22 participants), Unconcerned spenders cluster (two participants), and Comfort-oriented cluster (17 participants). Following Höpfl et al. (2024).

Linear mixed effect models (LMM) were conducted to test the proposed hypotheses for both temperature and cycle duration. They included feedback manipulations and sustainability clusters as fixed effects and participants as random effects. Due to a deviating aggregation level for cycle count, a repeated measures ANOVA was performed to inspect the effects of feedback and sustainability clusters as proposed in the hypotheses. All obtained effects were adjusted with Benjamini-Hochberg correction (Benjamini and Hochberg, 1995) to avoid type-I-error inflation.

Inspecting the effects of feedback manipulation on the dependent variables of temperature, cycle duration, and cycle count, analyses indicated a significant effect for temperature as displayed in Table 3, with a decrease in temperature by *M* = 0.4C for each condition. By contrast, no significant effect could be observed for cycle duration as outlined in Table 4 and cycle count (*F*(2, 126) = 0.21, *p* = 0.997, $\eta_{p}^{2}$ = 0.01). Hence, H1 can be partially confirmed in the tested sample.

**Table 3:** Regression table of the model with linear mixed effects for the variable temperature.

	**β**	***SE***	***z***	**95% CI**		***p***
				*LL*	*UL*	
Feedback	-0.38	0.13	-3.09	-0.65	-0.15	.006
Clusters	-0.67	1.25	-0.54	-3.12	1.78	.593
Feedback: Cluster	-0.05	0.05	-1.01	-0.15	0.05	.311

Number of observations = 2,086, number of subjects = 46, method: REML, Aikake Information Criterion (AIC) = 10,151.01, Bayesian Information Criterion (BIC) = 10,179.22, Log-Likelihood (LL) = -5,070.50, Intraclass Correlation Coefficient (ICC) = 0.96. CI, confidence interval; LL, lower limit; UL, upper limit. Adjustment of *p*-values with Benjamini-Hochberg correction (Benjamini and Hochberg, 1995). Skeptical consumer cluster (10 participants), Responsible saver cluster (22 participants), Unconcerned spenders cluster (two participants), and Comfort-oriented cluster (17 participants).

**Table 4:** Regression table of the model with linear mixed effects for the variable cycle duration.

	**β**	***SE***	***z***	**95% CI**		***p***
				*LL*	*UL*	
Feedback	3.00	2.324	1.29	-1.55	7.56	0.294
Clusters	6.3	3.931	1.60	-1.41	14.00	0.294
Feedback: Cluster	-0.91	0.88	-1.03	-2.64	0.82	0.304

Number of observations = 2,086, number of subjects = 46, method: REML, Aikake Information Criterion (AIC) = 22,048.23, Bayesian Information Criterion (BIC) = 22,076.44, Log-Likelihood (LL) = -111019.11, Intraclass Correlation Coefficient (ICC) = 0.34. CI, confidence interval; LL, lower limit; UL, upper limit. Adjustment of *p*-values with Benjamini-Hochberg correction (Benjamini and Hochberg, 1995). Skeptical consumer cluster (10 participants), Responsible saver cluster (22 participants), Unconcerned spenders cluster (two participants), and Comfort-oriented cluster (17 participants).

**Figure 4:**
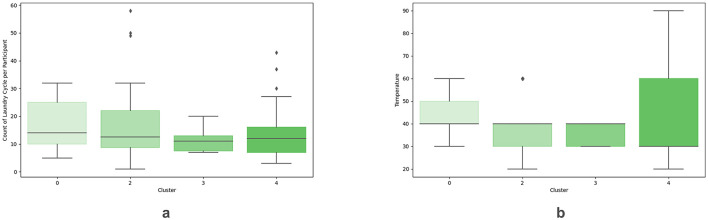
Boxplots of **(A)** the count of laundry cycles per participant per cluster, and **(B)** the temperature per cluster. 0 = Skeptical consumer cluster (10 participants), 2 = Responsible saver cluster (22 participants), 3 = Unconcerned spenders cluster (2 participants), and 4 = Comfort-oriented cluster (17 participants).

Examining the impacts of participants' assignment to different sustainability clusters on cycle count, temperature, and cycle duration indicates no significant differences, as presented for temperature in Table 3, for cycle duration in Table 4, and cycle count (*F*(3, 126) = 0.86, *p* = 0.997, $\eta_{p}^{2}$ = 0.02) as displayed in Table 2 and Figure 4. As a result, H2 could not be confirmed in the tested sample. To investigate if participants assigned to different sustainability clusters respond with different washing behaviors to feedback conveyed in EUR or kWh, we inspected the related interaction effects. Our analyses indicated no statistically significant interaction for temperature Table 3, cycle duration Table 4, or cycle count (*F*(6, 126) = 0.1, *p* = 0.997, $\eta_{p}^{2}$ = 0.00). Therefore, we could not receive support for H3 in our tested sample.

### 3.2 Qualitative results

From qualitative analyses of the interview transcripts, displayed in Table 5, we see that most participants wondered about the high energy savings from lower temperatures, their washing amount, the variability in electricity consumption for the same wash cycle, and the efficiency of the eco-program. According to participants' responses, different types of feedback partially influenced their washing behavior, while some aspects remained unchanged. Due to the low cost of a single wash cycle, some participants reported washing more carefree. Some participants were open to switching to a dynamic electricity price. A few participants experienced ease-of-use issues with the energy meter, while adjusting the electricity meter to their individual energy price was also reported to be a problem for some participants. Regarding technology improvements, many participants suggested changes in the time preselection function and further implementing feedback washing machines as present in the study. Participants expressed demand for an option to select the desired completion time for the washing machine instead of specifying when a washing cycle should start. Participants reported an according change would make it easier to schedule longer wash cycles, such as the eco-program which are harder to integrate in everyday life. Most participants expressed interest in gaining a better understanding of their electricity usage and the energy consumption associated with different wash cycles. Additionally, participants reported adopting new habits, such as using solar energy, the time preset function, setting an alarm clock, and eco-friendly washing cycles more often, leading to a more sustainable approach to washing.

**Table 5:** Interview excerpts from asynchronous interviews.

**Category**	**Quote**
Knowledge and interest increase for participants	•No.17 “When the condition with the kWh started, I was quite surprised at how much the consumption is when the washing temperature is lower. I found the difference (...) really immense and was surprised that there was such a big difference, even though it was only 10 degrees.” •No. 30 “I'm going to leave the appliance hanging and try a bit more.”
Behavior change	•No. 1 “No, I don't think it has changed.” •No.10 “(...) You could see how many more euro now, kWh, etc., were used. That gave me food for thought, and since then we've changed so that when I turn on the washing machine, I actually set a timer on my phone to remind me that the washing machine is ready and, ideally, I hang it up straight away.” •No. 20 “The feedback form has definitely changed my washing behavior. (...) this feedback form made me use it for the first time and I've actually used it more often since then. I think it's good because it gives me the opportunity to wash even when the electricity doesn't cost me anything, namely when the sun is shining.” •No. 32 “Generally no, except for the fact that I probably use this 40-degree eco cotton program more often, which is (...) longer. Otherwise, it hasn't really changed.”
Backfire effect	•No. 8 “I just have the feeling that I can wash more and it doesn't make me poor.” •No. 11 “I can simply start half a machine without worrying.”
Try out new programs	•No. 18 “The different forms of feedback (...) made me try out more.” •No. 30 “Now I've had some curious fun trying out what the individual programs run for and how they affect consumption at different temperatures.”
Swich to dynamic electricity price	•No. 1 “I would say yes, definitely. If there is potential for savings, we would definitely switch.”
Make it easier to wash sustainably, e.g., via technology	•No. 2 “Eco 40 to 60 degrees written down. That's simply the display for us. I can't see exactly what temperature it was set to in the end. That's why, for example, even when we were washing our baby clothes, we sometimes deliberately used the Hygiene+ program because we definitely wanted 60 degrees.” •No. 17 “(...) I would love smart networking with my cell phone. If I really get a reminder in the morning: Your washing machine is ready” •No. 20 “it would be great if this could be done via an app, e.g., where I could simply set the actual time or perhaps even be able to control it, (...) then I can start the laundry spontaneously.”
Feedback experiment	•No. 5 The euro condition “(...) was more difficult” (to adjust). •No. 32 “I must say the setup was easy for me. It was easy to connect. It was also very well thought out with the energy cost meter holder, so it wasn't lying around”
Satisfaction with prewash program	•No. 30 “I have a time-preset function in which I can preselect how many hours it should start. I would like to be able to specifically set the time at which it should be ready.”
Feedback on washing machine	•No. 22 “I think this information would be very interesting and useful if the washing machine could tell me how much this wash cycle has just cost me.” •No. 30 “I think a feedback module, which would be integrated into every washing machine from the outset, (...) whether with a green thumb or with a red, sad smiley or something like that, (...) it would make sense to integrate this as a standard function in the washing machine.”

## 4 Discussion

The increased GHG emissions and the resulting climate change endanger our modern societies, and private household energy consumption significantly contributes to these emissions. We aimed to analyze the effects of different types of feedback on individual washing behavior in a 9-week field experiment. Building on prior evidence we further investigated the impact of sustainability clusters, hinting that more personalized interventions could result in more sustainable behavior. We chose to focus on studying washing behavior because washing machines require limited interactions and can be operated by a single individual for the duration of the experiment, allowing us to measure this individual's behavior. Our quantitative results indicated partial support for our hypothesis within the selected sample. First, we proposed that participants would exert different washing behaviors if they received these different types of feedback (H1). Additionally, we expected participants assigned to different sustainability clusters to exert different washing behaviors (H2). Lastly, we assumed that participants assigned to different sustainability clusters would respond with different washing behaviors to feedback conveyed in EUR or kWh (H3). While we found significant differences between feedback conditions (H1) manifested in lower washing temperatures, we did not observe significant effects related to sustainability clusters (H2) or the interaction between feedback conditions and sustainability clusters (H3). The lack of significant results from (H2) and (H3) may be attributed to the small sample size used in the analysis. Moreover, it is conceivable that the intention-based screener is not jet capturing clusters that serve as accurate predictors of behavior. Exploring other screening criteria to improve the quality of the clusters might be beneficial. Additionally, participants qualitatively reported that they experienced habitual changes, felt more informed about the financial impact, and expressed a desire for a more accessible time-preset function. Our findings support the notion that sustainable behavior should be easy and cost-effective and promote habits to be exerted regularly. In the selected sample we found no support for the notion that behavior change interventions should be more individualized. The observed habitual changes in certain participants may indicate the need for a larger sample size to achieve more robust findings. Another possibility is that certain participants may be more amenable to behavior change interventions. The observed differences among individuals may provide insights into the potential for behavior change, potentially leading to the development of more effective approaches and identifiable clusters. From an applied perspective, our research holds implications for the design of products, services, and regulations by governments and companies. With the current pilot study, we provide reasonable justification to engage in subsequent larger-scale field investigations.

### 4.1 Implications

From a theoretical perspective, the key findings of our study support the effectiveness of accessibility, and habit formation strategies. Moreover, it is important to consider the potential impact of the feedback effect as a situational factor, in line with the theory of planned behavior (Ajzen, 1985). Feedback has the potential to mitigate the attitude-behavior gap by influencing situational factors. Moderators such as the green product availability and perceived consumer effectiveness can further leverage the attitude-behavior gap (Nguyen et al., 2019). The missing cluster effects do not support the notion of individualization for behavioral change strategies, implicating further research in this area.

Furthermore, most participants reported that they were adopting a more sustainable approach to washing and were willing to switch to a dynamic energy price if it would result in cost savings. A few participants reported washing more carelessly due to the new knowledge of the low cost of a single wash. While the long-term sustainability of these newly adopted habits remains a question, our qualitative findings generally support the argument that sustainable behaviors should be made more accessible. This claim is supported by significant effects on accessibility and habit formation, which were discovered in a meta-study on recycling behavior (Albarracín et al., 2024). Implementation of improved time preset function and enhanced transparency for the cost-benefit of ecological behavior should be a clear focus point for companies to prioritize encouraging users to change their behavior more effectively. Therefore, companies can address behavior change by shifting their design and innovation toward more sustainable products, services, and systems. In addition to reducing dependence on environmentally critical resources, the shift to a more sustainable design might be one of the most promising sustianability aspects. Companies can meet the growing demand for sustainable products by offering eco-friendly and socially responsible options. Hence, this might also raise the danger of greenwashing, which needs to be prevented by the companies and legislation. The shift toward sustainable design enables companies to gain competitive advantages and potentially expand their market share.

## 5 Limitations

On methodological accounts, *self-reported* studies can be inaccurate due to participant errors and influenced by various biases, such as social desirability bias. Given that washing machines were often located in basements without internet access, using an energy meter with an internet connection to automatically transmit data related to washing behavior to internal secure servers was not possible. Consequently, we were limited to using self-reported data instead of direct behavior monitoring.

In addition, the interviews conducted after the behavioral experiment highlighted issues with the chosen setup, including participants deviating from their regular washing behavior only due to the feedback conveyed by the energy meter. Besides, *potential backfire effects* occurred due to the newfound knowledge of cost-saving measures, as few participants considered the costs to be very low and reported washing more carelessly because of the study.

The clustering based on a decision tree, as reported in Höpfl et al. (2024), did not perform well on the current study's data. Upon closer inspection of the obtained pattern of results, the *number of subjects* for clustering might have impacted the quantitative part of the study, leading to an uneven distribution between clusters. Therefore, even if our sample is quite representative through the recruitment agency, a larger sample size might yield more impactful results.

Furthermore, our systematic clustering using a short *screening instrument* is based on theory-guided content about sustainable motivations and intentions, which has been validated by Höpfl et al. (2024) using a more extended survey. Nevertheless, the screening procedure is limited because it only contains a narrow set of scales and, therefore, cannot reflect reality, as is usual with shortened versions of longer inventories.

## 6 Future research

The previously outlined limitations could be addressed in a variety of subsequent experiments. When determining the required sample size for potential follow-up studies, it is crucial to ensure that emerging findings can be backed up in a statistically robust and generalizable manner. Therefore, a larger and more representative sample would help to reduce potential biases and strengthen the results, leading to more accurate and reliable conclusions. Since our research suggests that investigating the impacts of individual characteristics on behavior change is promising, future studies should enhance the methodological approach by utilizing a direct observation strategy. With an internet-connected web app, other household appliances such as the heating system could present an opportunity to further identify common characteristics among sustainable and unsustainable behavior clusters. Consequently, results might help to design and develop effective sustainable behavior change strategies. Additionally, an enhanced screening instrument to determine sustainability clusters seems promising. Since Albarracín et al. (2024) found significant results related to the social component a more explicit focus here could promising. For example, using a web application where the participant could compare their energy or cost-saving scores within their social community, might contribute additional insights into dynamics related to sustainable thought and action, with a possibility of shifting the perspective from individual consumers to services and communities such as households for behavior change interventions (Tchatchoua et al., 2023).

## 7 Conclusion

In conclusion, our qualitative results particularly highlighted accessibility, habit formation, and economic components as crucial contributing factors to promoting sustainable behavior. Hence, our quantitative findings demonstrate feedback as a promising strategy across the entire sample. Since participants assigned to different sustainability clusters did not respond with different washing behaviors we see the need for further studies in individualized behavior change interventions. Results of further studies might enable governments and companies to account for individual factors when assessing the systemic framework influencing consumer behavior. Nevertheless, we found feedback to be an effective behavior change intervention toward mitigating emissions, consequently alleviating the impact on the urgent matter of climate change.

## Data Availability

The datasets presented in this study can be found in online repositories. The names of the repository/repositories and accession number(s) can be found at: https://osf.io/un4e7/.

## Footnotes

^1^Our reported set of hypotheses differs from the preregistered hypothesis formulations to provide a more concise and coherent alignment with the chosen and preregistered research design.

^2^The preregistration, a comprehensive list of the included items, the qualitative questions, and additional information about recruitment procedures can be found in: https://osf.io/j3ny7.
